# Structure, Function, and Pharmaceutical Ligands of 5-Hydroxytryptamine 2B Receptor

**DOI:** 10.3390/ph14020076

**Published:** 2021-01-20

**Authors:** Qing Wang, Yu Zhou, Jianhui Huang, Niu Huang

**Affiliations:** 1 School of Pharmaceutical Science and Technology, Tianjin University, Tianjin 300072, China; wangqing@nibs.ac.cn (Q.W.); jhuang@tju.edu.cn (J.H.); 2 National Institute of Biological Sciences, No. 7 Science Park Road, Zhongguancun Life Science Park, Beijing 102206, China; zhouyu@nibs.ac.cn; 3 Tsinghua Institute of Multidisciplinary Biomedical Research, Tsinghua University, Beijing 102206, China

**Keywords:** GPCR, 5-HT_2B_R, biased signaling, agonist, antagonist

## Abstract

Since the first characterization of the 5-hydroxytryptamine 2B receptor (5-HT_2B_R) in 1992, significant progress has been made in 5-HT_2B_R research. Herein, we summarize the biological function, structure, and small-molecule pharmaceutical ligands of the 5-HT_2B_R. Emerging evidence has suggested that the 5-HT_2B_R is implicated in the regulation of the cardiovascular system, fibrosis disorders, cancer, the gastrointestinal (GI) tract, and the nervous system. Eight crystal complex structures of the 5-HT_2B_R bound with different ligands provided great insights into ligand recognition, activation mechanism, and biased signaling. Numerous 5-HT_2B_R antagonists have been discovered and developed, and several of them have advanced to clinical trials. It is expected that the novel 5-HT_2B_R antagonists with high potency and selectivity will lead to the development of first-in-class drugs in various therapeutic areas.

## 1. Introduction

5-Hydroxytryptamine (5-HT), or serotonin, was first isolated from beef serum and characterized in the late 1940s [1]. Biochemically, 5-HT is derived from the amino acid tryptophan, undergoing hydroxylation and decarboxylation processes that are catalyzed by tryptophan hydroxylase and aromatic L-amino acid decarboxylase, respectively [2]. As a biogenic amine, 5-HT plays important roles in cardiovascular function, bowel motility, platelet aggregation, hormone release, and psychiatric disorders [2]. 5-HT achieves its physiological functions by targeting various 5-HT receptors (5-HTRs), which are composed of six classes of G protein-coupled receptors (GPCRs) (5-HT_1_, 5-HT_2_, 5-HT_4_, 5-HT_5_, 5-HT_6,_ and 5-HT_7_ receptors, a total of 13 subtypes) and a class of cation-selective ligand-gated ion channels, the 5-HT_3_ receptor [3].

The 5-HT_2_ receptor (5-HT_2_R) subfamily is subdivided into 5-HT_2A_, 5-HT_2B_ and 5-HT_2C_ receptors. The 5-HT_2B_R was the last identified 5-HT_2_R family member and was first cloned in rat stomach fundus in 1992 [4], before the cloning of human 5-HT_2B_R in several tissues two years later [5,6]. In humans, the 5-HT_2B_R shares nearly 50% homology with the 5-HT_2A_R and 5-HT_2C_R, with about 70% homology in the transmembrane region [5]. Expressions of human 5-HT_2B_R mRNA have been detected in many different tissues, including the liver, kidney, intestine, pancreas, stomach, heart, lung, brain, uterus, trachea, testis, prostate, and placenta [5,6]. The 5-HT_2B_R is a G_q/11_ protein-coupled receptor. The activation of G_q/11_ results in several parallel signaling pathways. One branch of the canonical G_q/11_ signal transduction pathway is involved in the hydrolysis of guanosine triphosphate (GTP) to guanosine diphosphate (GDP) and is mediated by the G_q/11_ protein. The GTP-bound G_q/11_ stimulates the effector protein phospholipase Cβ (PLCβ) and leads to the generation of diacylglycerol (DAG) and inositol triphosphate (IP_3_), further increasing intracellular calcium ions and activating the protein kinase C (PKC) [7,8].

Significant progress has been made in the field of 5-HT_2B_R research in the past decade. Here, we review the recent updates of the biological functions, experimentally determined structures and pharmaceutical ligands of the 5-HT_2B_R, with a particular focus on clinical applications of 5-HT_2B_R antagonists. First, we elaborate on the important role that the 5-HT_2B_R plays in regulating the cardiovascular system, fibrosis disorders, cancer, the GI tract, and the nervous system. Second, we analyze the insights of the activation mechanism and biased signaling provided by the crystal structures. Finally, we summarize 5-HT_2B_R ligands that are clinically relevant or which have recently reported experimental verification data.

## 2. Function

### 2.1. Cardiovascular System

The 5-HT_2B_R is expressed in cardiovascular tissues, including myocardial, endothelial, and vascular smooth muscle cells [9]. Increasing evidence has revealed that the 5-HT_2B_R is involved in multiple cardiovascular diseases, including cardiomyopathy, valvular heart disease (VHD) and pulmonary arterial hypertension (PAH) [2,10].

#### 2.1.1. Cardiomyopathy

Since 2000, Nebigil et al. have suggested that the 5-HT_2B_R is implicated in regulating cardiac structure and function during embryogenesis and adulthood [9]. The ablation of the 5-HT_2B_R in mice led to embryonic and neonatal death. Surviving 5-HT_2B_R knockout mice exhibited cardiomyopathy with decreased cardiomyocyte number and size. On the contrary, specifically overexpressing the 5-HT_2B_R in the heart led to compensated hypertrophic cardiomyopathy, characterized by ventricular wall thickening [11].

Numerous animal model studies further confirmed the role played by the 5-HT_2B_R in cardiomyopathy. The 5-HT_2B_R has been found to be associated with isoproterenol- and noradrenaline-induced cardiac hypertrophy [12,13,14]. Chronic isoproterenol perfusion in mice imitating sympathetic stimulation induced cardiac hypertrophy, which could be prevented by treatment with 5-HT_2B_R antagonists, through regulating the hypertrophic cytokines produced by cardiac fibroblasts [12] and the production of superoxide anion [13]. In rats, a 5-HT_2B_R antagonist attenuated cardiac hypertrophy and myocardial apoptosis induced by chronic noradrenaline treatment [14]. In dogs with dilated cardiomyopathy, the 5-HT_2B_R was overexpressed in cardiomyocytes [15].

#### 2.1.2. VHD

The normal mammalian heart has four valves to ensure unidirectional blood flow during the cardiac cycle: the mitral valve (from the left atrium to the left ventricle), the tricuspid valve (from the right atrium to the right ventricle), the aortic valve (from the left ventricle to the aorta), and the pulmonary valve (from the right ventricle to the pulmonary artery). Any damaged or diseased heart valve can result in VHD. Abnormal valves cannot be fully open (stenosis) or fully close (regurgitation) so that the blood cannot be effectively pumped throughout the body, resulting in heart failure, sudden cardiac arrest and even death in more severe cases. Fully formed heart valves consist of valvular endothelial cells and valvular interstitial cells (VICs). The two types of cells regulate the generation of the extracellular matrix (ECM) and thus play critical roles in valve function [2]. Excessive ECM alters valve structure and leads to VHD.

Several drugs are known to associate with VHD side effects, including therapeutic agents for the treatment of obesity (fenfluramine and its stereoisomer dexfenfluramine, and benfluorex), Parkinson’s disease (pergolide and cabergoline), and migraine (methysergide and ergotamine), as well as the recreational drug 3,4-methylenedioxymethamphetamine (MDMA, commonly known as ecstasy) [16,17]. Drug-induced VHD has led to the withdrawal of fenfluramine and dexfenfluramine from the U.S. market in 1997, followed by the withdrawal of pergolide in 2007. Either these drugs or their metabolites have been demonstrated to be partial or full 5-HT_2B_R agonists with high affinity, and the pathogenesis of drug-induced VHD was correlated to the “off-target” activation of the 5-HT_2B_R [2,18]. Consequently, drug candidates with possible 5-HT_2B_R agonism effects are now required to be evaluated before approval [19]. Additionally, the signaling mechanism of drug-induced VHD has been studied [2,20]. Apart from the canonical G_q/11_ signal transduction pathway involved in the activation of PLCβ and PKC, the activation of the 5-HT_2B_R may also activate mitogenic pathways through the phosphorylation of the Src kinase and extracellular regulated kinases (ERK) and further enhance the activity of the transforming growth factor β (TGF-β). All pathways lead to VIC proliferation and ECM accumulation, and subsequently to the occurrence of VHD.

The 5-HT_2B_R was shown to be involved in vascular heart diseases, including mitral valve prolapse (MVP) [21] and calcific aortic valve disease (CAVD) [22,23]. Overexpression of the 5-HT_2B_R in the mitral valve leaflets was found in humans with MVP. Blockade of the 5-HT_2B_R mitigated mitral valve thickening and the activation of mitral valve interstitial cells, which are involved in the pathophysiology of MVP [21]. A study in isolated aortic valve interstitial cells (AVICs) in vitro showed that 5-HT_2B_R antagonism could prevent AVIC activation, a process associated with CAVD [22]. Recently, the same research group reported that in a high cholesterol diet mouse model, aortic valve hemodynamic development of CAVD could be attenuated by the ablation of the 5-HT_2B_ gene, but not 5-HT_2B_R antagonism [23].

#### 2.1.3. PAH

PAH is a progressive disorder characterized by abnormally high blood pressure in pulmonary arterial and pulmonary vasculature remodeling. The involvement of the 5-HT_2B_R in PAH has long been suggested. A significantly increased expression of the 5-HT_2B_R in pulmonary arteries was found in pulmonary hypertension (PH) patients and mice [24]. Moreover, the upregulation of the 5-HT_2B_R has been found in pulmonary artery smooth muscle cells derived from PAH patients [25]. In vivo studies on animal models suggested that chronic hypoxia or chemicals, such as deoxycorticosterone acetate (DOCA) salt and monocrotaline (MCT), could induce PH, which can be prevented or alleviated through blocking the 5-HT_2B_R or by genetic ablation [24,25,26,27]. In a BMPR2 mutant imitating heritable PAH mouse model, 5-HT_2B_R antagonism prevents PAH through reducing Src phosphorylation and downstream activity [28].

Emerging evidence has shown that bone marrow (BM)-derived cells contribute to 5-HT_2B_R-mediated PAH. Launay et al. found that lung cells overexpressing 5-HT_2B_Rs for vascular remodeling during PAH originate from BM precursors in mice [29]. They found that the specific expression of 5-HT_2B_R in the BM is necessary and sufficient for PAH development, whereas the ablation of 5-HT_2B_R on BM cells leads to resistance to PH. More recently, Bloodworth et al. demonstrated that BM-derived proangiogenic cells play a role in PH by mediating pulmonary arteriole stiffening and remodeling via the 5-HT_2B_R [30]. Both the ablation of BM-derived proangiogenic cells and 5-HT_2B_R antagonism prevented PH in mice with reductions in the number and stiffness of muscularized pulmonary arterioles.

### 2.2. Fibrosis Disorders

The 5-HT_2B_R has been implicated in fibrotic disorders such as liver fibrosis [31,32,33], pulmonary fibrosis [34,35,36,37,38], systemic sclerosis (SSc) [39,40,41,42,43], and pancreatic fibrosis [44].

#### 2.2.1. Liver Fibrosis

Liver fibrosis is generally believed to be caused by the excessive production of ECM, which is promoted by activated hepatic stellate cells (HSCs) transdifferentiating into myofibroblasts [45,46]. Ebrahimkhani et al. found that the 5-HT_2B_R was highly expressed in the diseased liver by activated HSCs and that 5-HT_2B_R antagonism exerted an antifibrogenic effect and improved liver function in a mouse model of progressive liver disease with fibrogenesis [31]. In a carbon tetrachloride (CCl_4_)-induced liver fibrosis mouse model, Li et al. found that chronic restraint stress alleviated liver fibrosis by inhibiting the activation of HSCs via the 5-HT_2B_R [32]. More recently, Xiang et al. revealed that two microRNAs (miR-221 and miR-222) were regulated by 5-HT during HSC activation and that the 5-HT_2B_R was essential for this regulation, as demonstrated by the discovery that 5-HT did not increase the expression of miR-221/miR-222 in 5-HT_2B_ knockdown HSCs [33].

#### 2.2.2. Pulmonary Fibrosis

Pulmonary fibrosis is one of the most studied 5-HT associated fibrosis. Fibroblasts (effector cells) differentiate into myofibroblasts and subsequently synthesize ECM, which are considered key events in pulmonary fibrogenesis. Fabre et al. found that the 5-HT_2B_R was highly expressed by fibroblasts in the fibroblastic foci in human idiopathic pulmonary fibrosis (IPF) samples [34]. In the lungs of IPF patients, Königshoff et al. found that the 5-HT_2B_R mainly localized to the epithelium and showed a significant increase in expression compared to transplant donors [35].

In vivo studies in the bleomycin (BLM)-induced pulmonary fibrosis mouse model suggested the involvement of 5-HT_2A_R and 5-HT_2B_R in pulmonary fibrosis. The expression of 5-HT_2A_R and 5-HT_2B_R were increased in the lung after the intratracheal treatment with BLM [34,35]. Blockade of the 5-HT_2A_R and 5-HT_2B_R could ameliorate BLM-induced lung fibrosis and improve lung function by reducing lung collagen content [34,35]. In vitro studies in human lung, fibroblasts showed that the antifibrotic effect of 5-HT_2A_R and 5-HT_2B_R antagonism was mediated by the TGF-β1 and WNT3α signaling pathways [35]. Moreover, Löfdahl et al. utilized two 5-HT_2B_R antagonists EXT5 and EXT9 (also with low to moderate affinity to the 5-HT_2A_/5-HT_2C_ receptors), to investigate the role of the 5-HT_2B_R in pulmonary fibrosis, suggesting their potential to prevent myofibroblast differentiation and subsequent fibrotic responses in a BLM-treated mouse model and human lung fibroblasts (see Section 4.2.1 for more details) [36]. Further studies suggested that the antiproliferative effects of EXT5 and EXT9 were related to the pAkt/p21 signaling pathway [38]. It is worth mentioning that in vivo studies in the BLM-induced pulmonary fibrosis rat model showed that 5-HT_2C_R and 5-HT_7_R were also implicated in pulmonary fibrosis [37,47,48].

#### 2.2.3. Systemic Sclerosis (SSc)

SSc is a chronic autoimmune disease characterized by progressive vascular disease and fibrosis of the skin and internal organs. Emerging evidence has suggested that 5-HT_2B_R plays an important role in SSc. In 2011, Dees et al. found that the expression of the 5-HT_2B_R was significantly increased in the skin of SSc patients compared with the normal skin of healthy individuals [39]. In vitro studies on SSc dermal fibroblasts suggested that the profibrotic effects of 5-HT are mediated by the 5-HT_2B_R, excluding the 5-HT_1B_R and 5-HT_2A_R, which are also expressed in dermal fibroblasts [39]. In vivo studies on BLM-induced dermal fibrosis and tight-skin-1 (tsk-1) mouse models showed that 5-HT_2B_R antagonists ameliorated fibrosis. In addition, mice lacking the 5-HT_2B_ gene could be protected from BLM-induced fibrosis [39]. In 2018, Chaturvedi et al. studied human adult dermal fibroblasts (HADF) isolated from SSc patients and showed that stimulation of 5-HT/TGF-β1 in HADF significantly increased the expression of profibrotic genes. Profibrotic genes were downregulated by the 5-HT_2B_R antagonist SB-204741, whose antifibrotic effect might be involved in the suppression of TGF-β1-mediated non-canonical (non-Smad dependent) signaling pathways [40]. Moreover, Wenglén et al. discovered selective antagonists of the 5-HT_2B_R (AM1125 and AM1476) and suggested their antifibrotic effects for the potential treatment of SSc (see Section 4.2.1 for details) [42,43].

### 2.3. Cancer

5-HT is involved in human cancer progression [49], and strong evidence has suggested that the 5-HT_2B_R plays a role in hepatocellular carcinoma (HCC), neuroendocrine tumor (NET) and pancreatic tumor.

#### 2.3.1. Hepatocellular Carcinoma (HCC)

Through the analysis of liver tissues from patients with HCC, Sarrouilhe et al. found that the 5-HT_1B_R and the 5-HT_2B_R were overexpressed in tumor tissues and that their antagonists inhibited proliferation of HCC cell lines, such as Huh7 and HepG2 [50]. Liang et al. suggested that 5-HT promoted the proliferation of serum-deprived Huh7 cells by upregulating the transcription factor FOXO3a, although this pro-proliferative effect was not observed in serum-deprived HepG2 or Hep3B cells [51]. They further found that the pro-proliferative effect of 5-HT could be blocked by the 5-HT_2B_R antagonist SB-204741 in Huh7 cells and that 5-HT_2B_R mRNA was significantly higher expressed in Huh7 cells compared to HepG2 and Hep3B cells, which may contribute to the distinct 5-HT effects in different serum-deprived HCC cells [51]. Using zebrafish HCC models, Yang et al. suggested that the 5-HT_2B_R was involved in HCC carcinogenesis [52,53]. In zebrafish, the expression of the 5-HT_2B_R was found to be high in HSCs, much lower in hepatocytes, and practically absent in neutrophils and macrophages [53]. The activation of the 5-HT_2B_R could increase both the proliferation and the activation of HSCs, as well as the expression of TGF-β1, resulting in liver enlargement and accelerating HCC carcinogenesis. In contrast, blocking the 5-HT_2B_R led to opposite effects [52,53].

#### 2.3.2. Neuroendocrine Tumor (NET)

NET is a rare type of tumor that most commonly arises in the GI tract and can lead to carcinoid syndrome [54,55]. Svejda et al. studied KRJ-I cells, a small intestinal-NET (SI-NET) cell line, and found that treatment with 5-HT_2B_R antagonist PRX-08066 inhibited the 5-HT secretion and KRJ-I cell proliferation, simultaneously decreasing the phosphorylation of ERK1/2 and the transcript levels and secretion of profibrotic growth factors, including TGF-β1, connective tissue growth factor (CTGF) and fibroblast growth factor (FGF2). The antiproliferative and antifibrotic effects of the 5-HT_2B_R suggested that this is a promising target for intervening SI-NETs [56].

#### 2.3.3. Pancreatic Tumor

In 2017, Jiang et al. reported that the 5-HT_2B_R could be used as a potential therapeutic target for intervening pancreatic ductal adenocarcinomas (PDACs) [57]. 5-HT was found to be increased in human PDAC tissues. Moreover, the incubation of 5-HT with PDAC cell lines resulted in an increase in PDAC cell proliferation and a decrease of PDAC cell apoptosis. Both in vitro and in vivo studies demonstrated that the pro-survival effect of 5-HT is mediated by the 5-HT_2B_R, but not other 5-HTRs. The 5-HT_2B_R agonist α-Me-HTP promoted the survival of PDAC cells, whereas the 5-HT_2B_R antagonist SB-204741 or genetic silencing of the 5-HT_2B_R blocked the pro-survival effect of 5-HT in PDAC cells and significantly reduced the tumor burden of PDAC in mice [57]. Moreover, the tumor-suppressive effects of 5-HT_2B_R antagonism were further confirmed in transgenic mice with pancreatic tumors. Notably, the mechanism behind 5-HT mediated PDAC cell survival involved the activation of PI3K/Akt/mTOR signaling and the enhancement of aerobic glycolysis (Warburg effect) [57].

### 2.4. Gastrointestinal (GI) Tract

Previous studies have suggested a role for the 5-HT_2B_R in the GI system. 5-HT_2B_R mRNA was widely expressed throughout the human GI tract [58]. The high expression of 5-HT_2B_R was detected in colonic smooth muscle, and the excitatory effects of 5-HT in the human colon were demonstrated to be mediated by the 5-HT_2B_R [58]. The 5-HT_2B_R was also found in the interstitial cells of Cajal (ICC), the “pacemaker cells” of the GI tract, which are expressed throughout the entire GI tract and required for normal GI motility. The activation of the 5-HT_2B_R in mouse models increased the proliferation of ICC in vitro and in vivo [59,60]. The 5-HT_2B_R triggered ICC proliferation was found to be mediated by PLC, intracellular calcium release and PKCγ [61].

Irritable bowel syndrome (IBS) is a common functional GI disorder that is characterized by abdominal discomfort and abnormal defecation. Visceral hypersensitivity is considered a hallmark characteristic of IBS. Many animal studies have demonstrated that the 5-HT_2B_R antagonism could help to modulate visceral hypersensitivity, colonic motility, and defecation [62,63,64,65], which indicates that the 5-HT_2B_R is a potential therapeutic target for GI disorders, especially for IBS. Notably, a study in conscious dogs showed that 5-HT_2B_R antagonism had no contractile effect on normal colonic motor activity and suggested that 5-HT_2B_R antagonists may be utilized for the treatment of diarrhea-predominant IBS without resulting in a constipation side effect [66].

### 2.5. Nervous System

As a neurotransmitter, 5-HT plays an essential role in the nervous system [67,68]. The 5-HT_2B_R has been suggested to mediate 5-HT functions in cognitive processes such as learning and memory [69,70,71], motor activities like breathing [72,73,74], as well as pain disorders, neuroglia function, and the dopaminergic pathway.

#### 2.5.1. Regulation of Pain Disorders

The 5-HT_2B_R has been implicated in migraine and neuropathic pain, which are two common forms of pain disorders in humans [75,76,77]. Migraine is a common primary headache disorder characterized by moderate to severe recurrent headaches. In 1989, Fozard et al. proposed that the initiation of migraine is caused by the activation of the 5-HT_2C_R [78]. However, this hypothesis was challenged after the cloning of rat 5-HT_2B_R in 1992 [4]. Subsequent studies demonstrated that the 5-HT_2B_R activation stimulated nitric oxide (NO) synthesis, which may be involved in migraine pathogenesis [75]. In guinea pigs, selective 5-HT_2B_R antagonists have been found to inhibit the 5-HT_2B_R/5-HT_2C_R agonist meta-chlorophenylpiperazine (mCPP) or the 5-HT_2B_R agonist BW723C686-induced dural plasma protein extravasation (PPE), an indicator for migraine attacks in animal models [79]. In addition, 5-HT_2B_R antagonism also prevented mCPP-induced dural PPE under hypoxia in mice [80].

Increasing evidence has revealed that the 5-HT_2B_R also plays a role in neuropathic pain [77]. In mouse dorsal root ganglion (DRG) neurons, the mechanical hyperalgesia induced by 5-HT or the 5-HT_2_R agonist α-m5-HT was inhibited by the 5-HT_2B_R/5-HT_2C_R antagonist SB-206553 [81]. Given that the 5-HT_2B_R was mainly expressed in DRGs, whereas the 5-HT_2C_R was detected only in trace amounts, 5-HT-induced mechanical hyperalgesia is most likely mediated by the 5-HT_2B_R [81]. Another signal transduction study suggested that the 5-HT_2B_R mediates the 5-HT-induced mechanical hyperalgesia through the PLCβ-PKCε pathway to regulate the function of transient receptor potential vanilloid 1 [82]. Cervantes-Durán et al. assessed the role played by peripheral and spinal 5-HT_2_Rs in formalin-induced secondary allodynia and hyperalgesia in rats. Local peripheral ipsilateral or intrathecal injection of selective 5-HT_2B_R antagonist significantly prevented formalin-induced nociceptive behavior monitored by flinching frequency [83]. Ipsilateral treatment with subtype-selective antagonists of 5-HT_2A_R, 5-HT_2B_R or 5-HT_2C_R, prevented formalin-induced long-term secondary mechanical allodynia and hyperalgesia [84]. Additionally, intrathecal treatment with the same antagonists inhibited formalin-induced long-term secondary mechanical allodynia and hyperalgesia in both ipsilateral and contralateral hind paws [85]. In the spinal nerve ligation-induced neuropathic pain rat model, intrathecal injection of 5-HT_2B_R antagonists not only impaired spinal nerve ligation-induced allodynia but also inhibited the spinal nerve injury-induced increased expression of the 5-HT_2B_R in both DRGs and spinal cord [86]. More recently, studies in female rats revealed that blocking the spinal 5-HT_2B_R diminished preoperative anxiety-induced postoperative hyperalgesia [87]. However, opposite findings were reported in other pain models. For example, in a rat model of neuropathic pain induced by chronic constriction injury (CCI) of the sciatic nerve, Urtikova et al. found that intrathecal injection of the 5-HT_2B_R agonist BW723C86 evidently relieved CCI-induced allodynia [88]. Clearly, further mechanistic studies are needed to explain the opposite experimental observations.

#### 2.5.2. Regulation of Neuroglia Function

The 5-HT_2B_R is also expressed in neuroglia, including microglia and astroglia, playing a role in regulating neuroglia function. Microglia, as the resident macrophages in the brain and the spinal cord, are responsible for the immune defense of the central nervous system (CNS) [89]. It was reported that the 5-HT_2B_R was expressed on postnatal microglia and participated in postnatal brain maturation [90]. More recently, the same group showed that the ablation of the 5-HT_2B_R gene in neonatal microglia was sufficient to cause enhanced weight loss and prolonged neuroinflammation in mice caused by exposure to lipopolysaccharides in adulthood. This suggested that the 5-HT_2B_R is required in neonatal microglia to prevent sickness behavior in adulthood [91].

Astrocytes are primary homeostatic cells of the CNS and account for about one-quarter of brain cortical volume. The expression of 5-HTRs, including the 5-HT_2B_R, has been found in both cultured and freshly isolated astrocytes [92]. Studies have suggested that conventional serotonin-specific reuptake inhibitors (SSRIs) such as fluoxetine act as agonists of astroglial 5-HT_2B_R [92]. The 5-HT_2B_R agonist BW723C86 could mimic the behavioral and neurogenic SSRI effects, which could be eliminated by the genetic or pharmacological inactivation of the 5-HT_2B_R [93]. In cultured mouse astrocytes, fluoxetine was found to induce EGFR transactivation and ERK1/2 phosphorylation, mediated by the stimulation of the 5-HT_2B_R [94], which is consistent with the observation that the drug-induced VHD involves the activation of the 5-HT_2B_R and consequent ERK phosphorylation [2,20]. Similarly, 5-HT was also found to cause ERK1/2 phosphorylation, which is mediated by the stimulation of the 5-HT_2B_R with high affinity and the 5-HT_2C_R with low-affinity [95]. Increasing evidence has suggested that astroglial 5-HT_2B_R is involved in depression [96]. In both 1-methyl-4-phenyl-1,2,3,6-tetrahydropyridine-induced and 6-hydroxydopamine-induced Parkinson’s disease mouse models [97,98], the decrease of astroglial 5-HT_2B_R expression paralleled the development of depressive behavior. Treatment with fluoxetine corrected both the decrease of astroglial 5-HT_2B_R expression and depressive behavior. All of these indicate that the downregulation of the astroglial 5-HT_2B_R may promote the development of depressive behavior in Parkinson’s disease. In addition, astroglial 5-HT_2B_R was also found to play a role in depressive behavior associated with sleep deprivation [99,100]. Specifically, the expression of the 5-HT_2B_R in a sleep deprivation mouse model was downregulated selectively in astrocytes, which was controlled by the activation of the P2X7 receptor [99]. Interestingly, leptin was found to increase the expression of astrocytic 5-HT_2B_Rs and thus enhance the action of fluoxetine on depressive-like behaviors induced by sleep deprivation [100].

#### 2.5.3. Regulation of the Dopaminergic Pathway

The 5-HT_2B_R has been implicated in the modulation of central dopamine (DA) activity, with potential applications in DA-dependent neuropsychiatric disorders, especially in schizophrenia and drug addiction [7,101].

Schizophrenia is a serious long-term mental disorder with multimodal symptomatology, characterized by positive, negative, and cognitive symptoms [102]. There is a classical hypothesis about schizophrenia proposed that positive symptoms are the result of a specific DA hyperfunction in the nucleus accumbens (NAc), whereas negative and cognitive symptoms are associated with a DA hypofunction in the medial prefrontal cortex (mPFC) [102]. Several microdialysis studies in rats suggested that the 5-HT_2B_R blockade exerts a differential control of DA ascending pathways, with increased, decreased and unaltered effects on DA outflow in the mPFC, the NAc, and the striatum, respectively. This is in accordance with the role played by DA neurotransmission in schizophrenia symptomatology [7,103,104,105]. An additional study indicated that the distinct effects caused by 5-HT_2B_R antagonists on mPFC and NAc DA outflow resulted from a functional interplay with mPFC 5-HT_1A_R [102]. Moreover, behavioral experiments in rats revealed that 5-HT_2B_R antagonists reduce phencyclidine-induced hyperlocomotion and reverse the phencyclidine-induced deficit in the novel object recognition test. These observations suggested that 5-HT_2B_R antagonists have the potential to alleviate the positive and cognitive symptoms of schizophrenia [105]. However, it was also reported that the ablation of the 5-HT_2B_R induces schizophrenic-like phenotypes, and contradictory results were observed in the DA outflow and behavior compared with 5-HT_2B_R antagonism in rats [106]. Hence, additional research studies are needed in order to explain the observed discrepancies and to confirm the role of the 5-HT_2B_R in the treatment of schizophrenia.

Several studies suggested that the 5-HT_2B_R may represent a potential pharmaceutical target for the treatment of drug addiction. From a behavioral point of view, blocking the 5-HT_2B_R may help to prevent MDMA-, amphetamine-, and cocaine-induced hyperlocomotion [103,104,107]. However, neurochemical responses vary according to different drugs. For example, 5-HT_2B_R antagonists inhibit MDMA- and amphetamine-induced DA outflow in the NAc [103,107], but no effects were observed on cocaine-induced DA outflow in the NAc [104]. Recent findings showed that the dorsal raphe nucleus 5-HT_2B_R blockade suppresses cocaine-induced hyperlocomotion resulting from the facilitation of mPFC DA outflow, which would subsequently inhibit accumbal DA neurotransmission [108].

## 3. Structure

### 3.1. Crystal Structures

The 5-HT_2B_R belongs to the class A GPCRs, the largest subfamily of GPCRs, and shares a conserved architecture: seven transmembrane helices (I-VII) followed by an 8th helix (VIII), three extracellular loops (ECL1–ECL3), three intracellular loops (ICL1–ICL3), an extracellular N-terminus and an intracellular C-terminus [109]. The 5-HT_2B_R contains 481 amino acids with a molecular mass of about 54.3 kDa.

**Figure 1:**
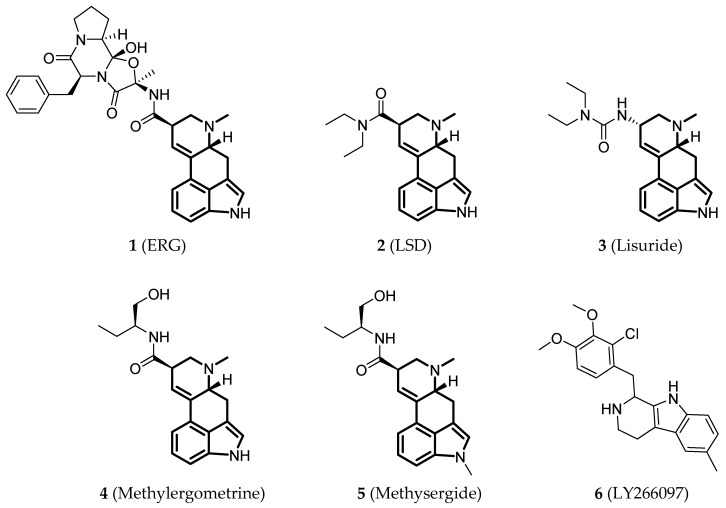
Small-molecule ligands in co-crystal structures of 5-HT_2B_R-ligand complexes.

Since the 5-HT_2B_R crystal structure was first determined in 2013 [110], a total of eight crystal complex structures of the 5-HT_2B_R bound with small-molecule ligands were published to date (Table 1). These include five representative ergolines (ergotamine (ERG, **1**), lysergic acid diethylamine (LSD, **2**), lisuride (**3**), methylergometrine (**4**, also called methylergonovine), methysergide (**5**)), and one selective antagonist of the 5-HT_2B_R (LY266097 (**6**)) (Figure 1) [110,111,112,113,114]. These ergolines are promiscuous ligands for many types of aminergic GPCRs and possess distinct functions. For example, despite ERG and LSD are both β-arrestin-biased agonists of the 5-HT_2B_R, ERG has antimigraine effects whereas LSD is hallucinogenic; lisuride is structurally similar to LSD, but it shows antagonistic effects towards the 5-HT_2B_R; methysergide is an antagonist of the 5-HT_2B_R with an antimigraine effect that in vivo it rapidly undergoes N-demethylation and transforms into methylergometrine, a potent agonist of the 5-HT_2B_R. In one crystal structure (PDB ID: 5TUD), the 5-HT_2B_R binds with ERG and an antibody Fab fragment on its extracellular side in an active-like conformation [113]. Taken together, the structural information provides an unprecedented opportunity to understand the ligand recognition, activation mechanism and biased signaling.

### 3.2. Conformational States

**Figure 2:**
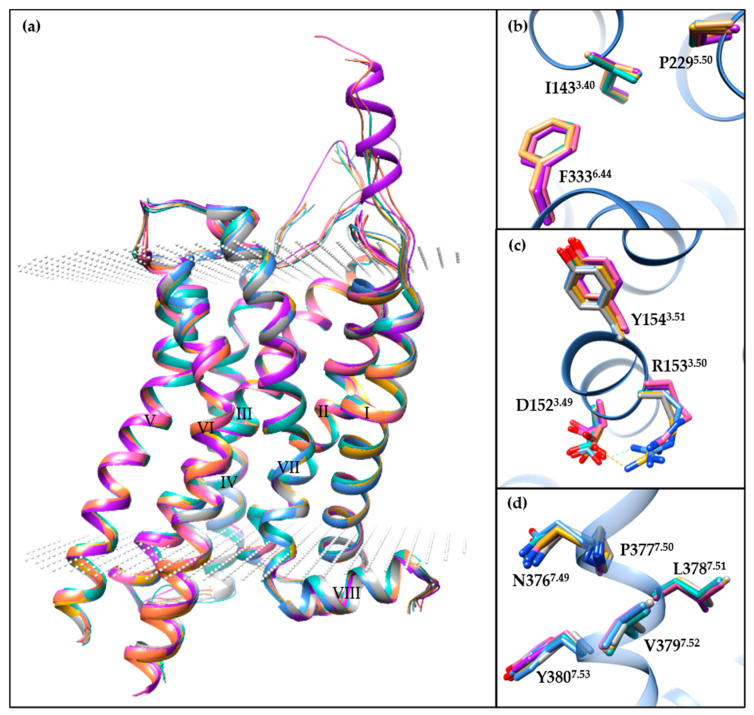
A comparison of the different small-molecule-bound 5-HT_2B_R crystal structures. (**a**) The overall architecture of 5-HT_2B_R/ERG (cornflower blue, PDB ID: 4IB4), 5-HT_2B_R/ERG (dark gray, PDB ID: 4NC3), 5-HT_2B_R/LSD (hot pink, PDB ID: 5TVN), 5-HT_2B_R/lisuride (light sea green, PDB ID: 6DRX), 5-HT_2B_R/methylergometrine (salmon, PDB ID: 6DRY), 5-HT_2B_R/methysergide (purple, PDB ID: 6DRZ), 5-HT_2B_R/LY266097 (goldenrod, PDB ID: 6DS0) structures aligned through the Cα atoms of residues in helices I to VIII. The 5-HT_2B_Rs are displayed as ribbon cartoons, and the membrane boundaries are displayed as white dots, according to the Orientations of Proteins in Membranes database. (**b**–**d**) Specific comparison of residues in (**b**) PIF, (**c**) D(E)RY and (**d**) NPxxY motifs. For clear visualization, residues apart from the motif are hidden, with the exception of the 5-HT_2B_R/ERG structure (PDB ID: 4IB4). Molecular images were generated using the UCSF Chimera software [115].

Apart from the 5-HT_2B_R/ERG-Fab structure (PDB ID: 5TUD), the other seven small-molecule-bound crystal structures show similar conformations (Figure 2a), especially in the helical region. Specifically, the Cα root-mean-square deviation (RMSD) values range from 0.34 to 0.60 Å using the 5-HT_2B_R/ERG structure as the reference (PDB ID: 4IB4). For the most classical microswitches in class A GPCRs, the seven structures show extremely similar conformations in PIF (Figure 2b) and NPxxY (Figure 2d) motifs and a slightly different conformation in the D(E)RY motif (Figure 2c). The salt bridge in the D(E)RY motif between D152^3.49^ and R153^3.50^ can only be observed in two of the structures (PDB IDs: 4IB4 and 6DS0). The electron densities for R153^3.50^ side chains in four other structures are not well-defined (PDB IDs: 4NC3, 6DRX, 6DRY, and 6DRZ), and the D152^3.49^ side chains are in different orientations. An analysis of the B-factor distributions suggests that the conformations of D152^3.49^ and R153^3.50^ side chains are unstable with relatively large B-factor values.

**Figure 3:**
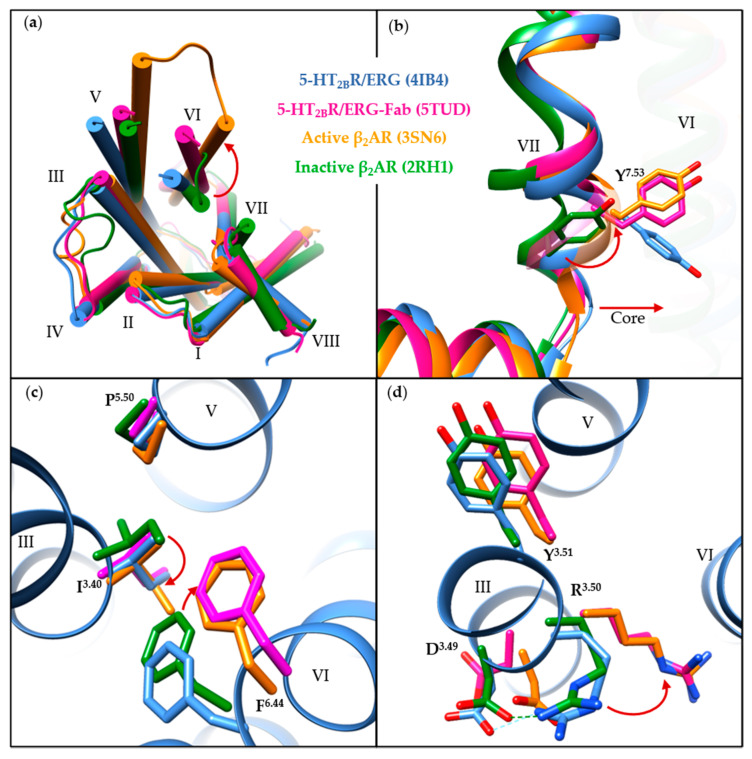
A comparison of the 5-HT_2B_R/ERG (cornflower blue, PDB ID: 4IB4), the 5-HT_2B_R/ERG-Fab (magenta, PDB ID: 5TUD), the active-state β_2_AR (orange, PDB ID: 3SN6) and the inactive-state β_2_AR (forest green, PDB ID: 2RH1) crystal structures. (**a**) Intracellular view of the overall architecture. The outward movement of helix VI upon activation is indicated by a red arrow. (**b**) A comparison of structural features in helix VII. The slight inward movement of helix VII and rotation of Y^7.53^ in the NPxxY motif towards helix VI are highlighted by red arrows. (**c**,**d**) A comparison of the residues in (**c**) PIF and (**d**) D(E)RY motifs. For clear visualization, residues apart from the motif are hidden, with the exception of the 5-HT_2B_R/ERG structure (PDB ID: 4IB4). The obvious conformational changes of specific residues upon activation are shown as red arrows. Molecular images were generated using the UCSF Chimera software [115].

5-HT_2B_R/ERG-Fab (PDB ID: 5TUD) structure shows distinct features compared with the other seven structures. Since the determined small-molecule-bound structures share common structural features in the transmembrane regions and the key motifs, we compared the 5-HT_2B_R/ERG structure (PDB ID: 4IB4) with the 5-HT_2B_R/ERG-Fab structure in order to illustrate the conformational changes of the 5-HT_2B_R, using the well-known active-state (PDB ID: 3SN6) [116] and inactive-state (PDB ID: 2RH1) [117] β_2_-adrenergic receptor (β_2_AR) structures as references (Figure 3a). Upon activation, an outward movement of the intracellular helix VI (Figure 3a), and an inward shift of helix VII along with a side-chain rotation of Y^7.53^ (Figure 3b), are believed to represent common features in class A GPCRs [118,119]. When compared to the 5-HT_2B_R/ERG structure, the 5-HT_2B_R/ERG-Fab structure shows a more evident outward movement of the intracellular helix VI (Figure 3a). The backbone atoms of helix VII in both the 5-HT_2B_R/ERG and the 5-HT_2B_R/ERG-Fab structures overlapped well with the active-state β_2_AR structure, whereas the orientation of the Y^7.53^ side chain in 5-HT_2B_R/ERG is slightly different (Figure 3b). In the PIF motif, the three residues in the 5-HT_2B_R/ERG-Fab structure show active-like conformations. Although the P229^5.50^ and the I143^3.40^ residues show active-like conformations in the 5-HT_2B_R/ERG structure, the F333^6.44^ residue shows an inactive-like conformation (Figure 3c). In the D(E)RY motif, the R153^3.50^ in the 5-HT_2B_R/ERG-Fab structure displays an extended conformation towards helix VI, similar to the active-state β_2_AR structure. Hence, the salt bridge between D152^3.49^ and R153^3.50^ is fully broken (Figure 3d). In summary, considering the helical movement and microswitches, the 5-HT_2B_R/ERG-Fab structure shows an active-like state, whereas the 5-HT_2B_R/ERG and other small-molecule bound 5-HT_2B_R structures are in intermediate states.

### 3.3. Orthosteric Binding Pocket (OBP)

**Figure 4:**
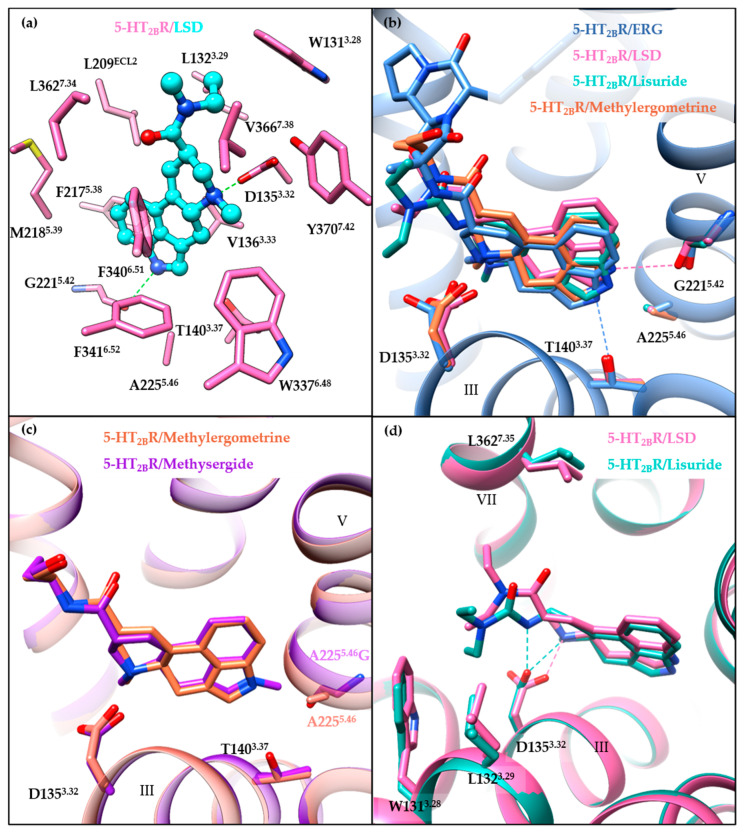
Overview of receptor–ligand interactions in the binding pocket. (**a**) Receptor–ligand interactions in the binding pocket of the 5-HT_2B_R/LSD structure (PDB ID: 5TVN). Residues of the 5-HT_2B_R are colored in hot pink, and LSD is colored in cyan. (**b**) A comparison of the specific interactions in the OBP of ergoline ligand-bound structures: 5-HT_2B_R/ERG (cornflower blue, PDB ID: 4IB4), 5-HT_2B_R/LSD (hot pink, PDB ID: 5TVN), 5-HT_2B_R/lisuride (light sea green, PDB ID: 6DRX), and 5-HT_2B_R/methylergometrine (salmon, PDB ID: 6DRY). For clear visualization, only the backbone of 5-HT_2B_R/ERG is shown as a ribbon cartoon. The hydrogen bond interactions are displayed as dashed lines. (**c**) Comparison of the binding mode of the 5-HT_2B_R/methylergometrine (salmon, PDB ID: 6DRY) and the A225^5.46^G mutant 5-HT_2B_R/methysergide (purple, PDB ID: 6DRZ) structures. (**d**) Comparison of the specific interactions in the EBP of the 5-HT_2B_R/LSD (hot pink, PDB ID: 5TVN) and the 5-HT_2B_R/lisuride structures (light sea green, PDB ID: 6DRX). Hydrogen bond interactions are shown as dashed lines. Molecular images were generated using the UCSF Chimera software [115].

All of the small-molecule ligands in the published 5-HT_2B_R co-crystal structures occupy the OBP that is presumed as the 5-HT’s binding pocket, as well as regions outside the OBP and close to the extracellular loops, termed as the extended binding pocket (EBP) [120]. Taking LSD’s binding mode as an example, the ergoline ring system occupies the OBP by interacting residues in helices III, V, VI, and VII (Figure 4a). These interactions are commonly observed in aminergic GPCRs structures, including a conserved salt bridge between the positively charged nitrogen of the ligand and the carboxylic acid of D^3.32^, and π-π stacking interactions formed by the aromatic system of the ligand with residues F^6.51^ and F^6.52^ in helix VI. In the EBP, the diethylamide moiety of LSD interacts with residues in the extracellular side of helices III and VII as well as ECL2.

A comparison of the binding modes of ergoline ligands (ERG, LSD, lisuride, and methylergometrine) shows subtle differences (Figure 4b). Different orientations of the ergoline scaffolds seem to be caused by the different ergoline substituents, along with the rotation of D135^3.32^ side chains and the different orientations of the indole nitrogens towards helix V residues. The indole nitrogen atom of LSD points towards the backbone carbonyl oxygen of G221^5.42^, whereas the indole nitrogen atoms of ERG, lisuride, and methysergide point towards the A225^5.46^ residue and form hydrogen bonds (for ERG) or electrostatic interactions (for lisuride and methysergide) with the hydroxyl oxygen of L362. In fact, both T140^3.37^ and A225^5.46^ have been suggested to play significant roles in the activation mechanism [114]. On one hand, the T140A^3.37^ mutant substantially disrupted methylergometrine’s G_q_ agonism and β-arrestin2 recruitment. On the other hand, the A225G^5.46^ mutant restored both methysergide’s G_q_ agonism and β-arrestin2 recruitment. Moreover, the crystal structure of methysergide-bound 5-HT_2B_R with A225G^5.46^ mutation further explained the methysergide antagonism. Methysergide adopts a similar binding mode with methylergometrine in the 5-HT_2B_R/methylergometrine structure (Figure 4c), and its N-methyl group (which is the only difference between the two ligands) is oriented towards the mutated G225^5.46^ residue, suggesting that the A225G^5.46^ mutant results in larger space accommodate the ligands. Thus, pushing outward on helix V may be responsible for methysergide’s antagonism. Hence, both A225^5.46^ and T140^3.37^ are key residues in the OBP for the 5-HT_2B_R activation with equal contributions for G_q_ and β-arrestin2 activation mechanisms.

### 3.4. Extended Binding Pocket (EBP)

The role played by the EBP in receptor activation has been illustrated by comparing chemically similar but functionally distinct compounds such as LSD (5-HT_2B_R agonist) and lisuride (5-HT_2B_R antagonist). LSD and lisuride bear different substitutions on the ergoline scaffold, containing an (*R*)-diethylamide and an (*S*)-diethylurea substitution, respectively (Figure 1). Therefore, the main differences between their binding modes arise from the EBP. Lisuride’s (*S*)-diethylurea points towards helix III, forming hydrophobic interactions with W131^3.28^ and L132^3.29^, and an additional hydrogen bond with D135^3.32^ (Figure 4d). While LSD’s (*R*)-diethylamide interacts with helices III and VII, and forms additional interactions with L362^7.35^ in helix VII. Consequently, contact with helix VII appears to be essential for 5-HT_2B_R activation. A mutagenesis experiment of the L362^7.35^ residue in the 5-HT_2B_R has highlighted its crucial role in receptor activation, as mutants facilitating the contact with helix VII (L362N^7.35^, L362F^7.35^, L362Y^7.35^) restored lisuride’s G_q_ agonism. On the contrary, L362A^7.35^ mutant impaired LSD’s G_q_ agonism [114].

Moreover, the ligand recognition at helix VII in the EBP was also suggested to play an essential role in biased signaling. The L362F^7.35^ mutant restored lisuride’s G_q_ agonism, but not β-arrestin2 recruitment agonism [114]. For LSD, the L362F^7.35^ mutant abolished LSD’s β-arrestin2 recruitment without affecting its G_q_ agonism [114]. LY266097, a selective 5-HT_2B_R antagonist with modest G_q_ partial agonism and potent β-arrestin2 antagonism, interacts with residues in helix VII, including L362^7.35^. LY266097’s agonist potency was abolished in the L362F^7.35^ mutant [114]. Moreover, structure–activity relationship (SAR) studies showed that an analog of LY266097 lacking substituents on the benzyl moiety was able to fully abolish its G_q_ agonism [114].

Notably, residues in ECL2 have also been indicated to play a role in LSD’s long residence time and biased signaling [112]. Molecular dynamics (MD) simulation results suggested that the fluctuation of the “lid” in ECL2 (residues 207–214) may influence LSD’s dissociation and that the L209^ECL2^A mutant may act by increasing the flexibility of the lid [112]. Further experimental verification found that the L209^ECL2^A mutant decreased LSD’s residence time and significantly reduced the β-arrestin2 recruitment potency and efficacy without affecting G_q_ agonism [112]. In addition, LSD’s β-arrestin2 recruitment was found to be time-dependent for both the 5-HT_2A_R and the 5-HT_2B_R. The L229^ECL2^A and L209^ECL2^A mutants could selectively abolish the 5-HT_2A_R’s, and the 5-HT_2B_R’s time-dependent β-arrestin2 recruitment, respectively [112].

In summary, key residues have been suggested to play important roles in receptor activation and biased signaling. Ligand recognition at helices III (T140^3.37^) and V (G221^5.42^, A225^5.46^) in the OBP appear to contribute equivalently to G_q_ and β-arrestin2 potency, whereas ligand recognition at helix VII (L362^7.35^) and ECL2 (L209^ECL2^) contributes to either G_q_ or β-arrestin2 activity. All of these would guide the discovery of novel 5-HT_2B_R ligands, especially for biased ligands. It is expected that biased ligands may provide more possibilities for enhancing therapeutic efficiencies and reducing adverse effects [121]. For example, a β-arrestin biased antagonist of dopamine D2 receptor (BRD5814) was shown to have considerable antipsychotic efficacy and reduced motoric side effects in a mouse model [122].

## 4. Pharmaceutical Ligands

As 5-HT_2B_R agonism has been considered as a side effect related to VHD, pharmaceutical ligand discovery has focused on the potential application of 5-HT_2B_R antagonism. Considering that many 5-HT_2B_R ligands early identified have been previously summarized [123,124], we mainly focus on reviewing the clinical-related pharmaceutical ligands and promising antagonists of the 5-HT_2B_R that were reported since 2010. Note that the *K*_i_ values mentioned below were determined by radiobinding assays.

### 4.1. Clinical-Related Pharmaceutical Ligands of the 5-HT_2B_R

#### 4.1.1. MT-500

MT-500 (**7**, RS-127445, Table 2) is a 5-HT_2B_R antagonist with high affinity (*K*_i_ = 0.3 nM) and high selectivity over many other 5-HT receptor subtypes (especially about 1000-fold selectivity over the closely related human 5-HT_2A_R and 5-HT_2C_R) [125]. In vitro functional assays demonstrated that MT-500 could inhibit the 5-HT-induced increases of inositol phosphates and intracellular calcium concentration (IC_50_ = 0.1 nM) and block the 5-HT-induced contraction of the rat stomach fundus [125]. In addition, in vivo pharmacokinetic studies in rats showed that MT-500 was readily absorbed by oral or intraperitoneal routes [125]. Several studies used MT-500 as a tool compound to investigate the functions of the 5-HT_2B_R [62,63,83,85,108]. In 1999, POZEN acquired MT-500 from Roche and took full charge of its development for migraine prophylaxis. The phase 1 clinical trial of MT-500 was completed and showed an encouraging safety profile. However, the development of MT-500 was discontinued in 2002 for unknown reasons.

#### 4.1.2. PRX-08066

PRX-08066 (**8**, Table 2) is a potent (*K*_i_ ~ 3.4 nM) and selective antagonist of the 5-HT_2B_R discovered by Porvasnik et al. [126]. In the study using the MCT-induced PAH rat model, PRX-08066 eased the severity of PAH by significantly reducing the elevation in PA pressure and right ventricle hypertrophy [126].

In May 2005, PRX-08066 entered a phase 1 clinical trial. The results of a dose-escalation study in healthy volunteers indicated that PRX-08066 was well-tolerated over a dose ranged from 25 mg to 800 mg, and no serious adverse events were observed. Later, PRX-08066 phase 1b safety and pharmacodynamic studies in healthy athletes started in November 2005. When volunteers took 200 mg of PRX-08066 orally, the increase in pulmonary artery blood pressure was significantly reduced (by 40%) during hypoxic exercise and did not influence systemic blood pressure. PRX-08066 thus advanced into phase 2 clinical trials in June 2006 (NCT00345774). The short-term safety and efficacy of PRX-08066 were evaluated in patients with PH associated with chronic obstructive pulmonary disease (COPD). The results were positive and indicated that PRX-08066 could significantly lower systolic pulmonary arterial pressure and was well-tolerated without any obvious effect on systemic blood pressure. In August 2008, PRX-08066 open-label phase 2 clinical trials commenced in order to assess its safety and efficacy in PH and COPD patients (NCT00677872). However, the trial was also later terminated, with no results reported.

#### 4.1.3. BF-1

BF-1 (**9**, Table 2) is a selective and high-affinity antagonist of the 5-HT_2B_R (*K*_i_ = 2.7 nM) [79]. Tested in a guinea pig model for dural neurogenic PPE induced by mCPP or BW723C86, BF-1 exhibited significant reductions of dural PPE, indicating its potential as a migraine drug [79]. In December 2012, BF-1 commenced phase 1 clinical trials prophylactic for the treatment of migraine. However, no progress has since been reported, and the clinical trial was postulated to be discontinued.

#### 4.1.4. AMAP102

AMAP102 (**10**, Table 2) is an orally available antagonist of the 5-HT_2B_R (structure and binding affinity are undisclosed) [127]. In vitro studies showed that AMAP102 decreased the release of proinflammatory cytokine TNF-α in human macrophages and IL-6 in rat synovial fibroblasts [127]. In vivo studies on several animal models, including collagen and glucose-6-phosphate isomerase-induced arthritis induced arthritis in mice and antigen-induced arthritis in rats, showed that AMAP102 exhibited anti-arthritic effects and reduced inflammatory pain responses [127].

In April 2009, AMAP102 entered a phase 1 clinical trial (NCT00995605) to evaluate its safety and tolerability in healthy subjects. The trial was successfully completed in August 2009, and the results showed that AMAP102 was safe and well-tolerated without any serious adverse events. In October 2014, AnaMar AB company reported the phase 2a results of AMAP102 for the treatment of inflammatory pain in osteoarthritis patients. However, compared with placebo, AMAP102 did not show a statistically significant reduction in pain over a 28-day period, and the AMAP102 clinical trial was thus discontinued.

#### 4.1.5. AM1030

AM1030 (**11**, Table 2) [128], an aminoguanidine derivative (structure is not disclosed), is a 5-HT_2B_R antagonist (*K*_i_ = 330 nM). In vivo and in vitro studies on various human and rodent models suggested that AM1030 has therapeutic potential in various inflammatory diseases and is able to significantly reduce both T cell-dependent and T cell-independent inflammatory responses. Moreover, a first-in-man study in atopic dermatitis (AD) patients showed that topical administration of AM1030 was suitable for treatment. AM1030 phase 1/2 clinical trials studied in AD patients were completed in June 2015 (NCT02379910), but the results were not reported. Considering no development has since been disclosed, the development of AM1030 is assumed to be discontinued.

#### 4.1.6. RQ-00310941

RQ-00310941 (**12**, Table 2) is an antagonist of the 5-HT_2B_R with high affinity (*K*_i_ = 2.0 nM) and good selectivity (>2000-fold selectivity against more than 60 GPCRs, ion channels, and enzymes) [64]. In vivo studies in the trinitrobenzene sulfonate-induced visceral hypersensitivity rat model indicated that RQ-00310941 has the therapeutic potential for diarrhea-predominant IBS [64]. Specifically, RQ-00310941 could attenuate the distal colon sensitivity (61% inhibition at 1 mg/kg, per os (p.o.)) and suppress the restraint stress-induced defecation (95% inhibition at 10 mg/kg, p.o.) without affecting normal defecation at high dose (30 mg/kg, p.o.) [64].

In July 2015, RQ-00310941 entered phase 1 clinical trials. Specifically, the safety/tolerability and pharmacokinetics in healthy subjects and the preliminary efficacy in mild to moderate ulcerative colitis (UC) patients with IBS-like symptoms were investigated [129]. The released results showed that RQ-00310941 was safe and well-tolerated without any serious adverse events in both healthy subjects and UC patients. Although there was no statistically significant difference observed between RQ-00310941 and placebo with respect to primary and secondary efficacy evaluations, most efficacy measures suggested slightly favorable outcomes compared to placebo, indicating a therapeutic potential for RQ-00310941 in the treatment of IBS-like symptoms in UC patients.

### 4.2. Representative Pharmaceutical Ligands of the 5-HT_2B_R

#### 4.2.1. EXT5, EXT9, AM1125, and AM1476

AnaMar AB company is focused on the discovery and development of 5-HT_2B_R antagonists to prevent pathological inflammatory and fibrotic processes. Apart from AMAP102 (**10**) (Section 4.1.4) and AM1030 (**11**) (Section 4.1.5), which were mentioned above, they have also developed EXT5 (**13**), EXT9 (**14**), AM1125 (**15**), and AM1476 (**16**). The exact structures of these compounds are undisclosed. EXT5 and EXT9 are both benzylidene aminoguanidine derivatives [36], while AM1125 and AM1476 have general formula containing a 1-amidino-3-aryl-2-pyrazoline scaffold [130].

EXT5 (**13**) and EXT9 (**14**) are both mainly antagonizing the 5-HT_2B_R (EXT5: *K*_i_ = 45 nM, IC_50_ = 82 nM in IP1 accumulation assay; EXT9: *K*_i_ = 26 nM, IC_50_ = 29 nM in IP1 accumulation assay), and also exhibit low to moderate binding affinities to the 5-HT_2A_R and the 5-HT_2C_R. As mentioned above (Section 2.2.2), EXT5 and EXT9 were utilized for investigating the role played by the 5-HT_2B_R in fibrosis [36]. In vitro, the co-cultivation of TGF-β1 and 5-HT resulted in an increased α-SMA and proteoglycan production, which could be significantly decreased after the treatment with either EXT5 or EXT9. Additionally, in vivo studies on BLM-treated mice showed that both EXT5 and EXT9 could attenuate the fibrotic tissue remodeling, demonstrated by a decrease in tissue density, collagen-producing cells, myofibroblasts, and decorin expression [36]. Further gene expression studies suggested that the antiproliferative effects of EXT5 and EXT9 may be associated with the pAkt/p21 signaling pathway, a cell-cycle regulation pathway [38]. More recently, EXT5 and EXT9 were also used for investigating the role played by the 5-HT_2B_R on airway function and remodeling [131]. Studies showed that EXT5 and EXT9 inhibited 5-HT-induced bronchoconstriction, TGF-β1 release and the proliferation of smooth muscle cells [131]. Notably, the 5-HT-induced bronchoconstriction could also be suppressed by the 5-HT_2A_R/5-HT_2C_R antagonist ketanserin, but not by the 5-HT_2B_R selective antagonists RS-127445 or PRX-08066 [131]. The inhibitory effects of bronchoconstriction may involve a combination of 5-HT_2_ receptors and deserves further research efforts in the future.

AM1125 (**15**) is a highly selective 5-HT_2B_R antagonist (*K*_i_ = 0.9 nM). In November 2016, preclinical data of AM1125 was presented at the ACR annual meeting [132]. Treatment with AM1125 at 50 mg/kg reduced fibrosis parameters (hypodermal thickness, myofibroblast counts, and hydroxyproline content) in the tsk-1 model of SSc, which indicated a potential treatment opportunity for SSc. In May 2017, preclinical data on the antifibrotic effects of AM1125 was reported at the ATS International Conference [42]. In in vitro studies on human lung, fibroblasts showed that treatment with AM1125 significantly reduced TGF-β mRNA, plasminogen activator inhibitor 1 mRNA, and phosphorylated Smad 2/3. Further in vivo studies on BLM-induced pulmonary fibrosis mice showed that the oral administration of AM1125 ameliorated pulmonary fibrosis with a reduction of the fibrotic area, myofibroblast counts and the amount of collagen protein. These results reflected the potential of AM1125 for the treatment of pulmonary fibrosis.

AM1476 (**16**), an orally available 5-HT_2B_R antagonist with high selectivity (activity data are undisclosed), is currently in the late preclinical phase and under development for SSc [43]. Orally administration of AM1476 in the murine sclerodermatous chronic graft-versus-host disease model showed that it could significantly reduce all measured dermal and pulmonary fibrosis readouts [43]. Dermal fibrosis studied in the tsk-1 model of SSc showed reduced hypodermal thickening and the number of myofibroblast and hydroxyproline content. In addition, the number of pSmad3 positive cells was significantly reduced in skin samples suggested the inhibitory effect on the TGF-β/Smad signaling pathway [43].

#### 4.2.2. Bis-Amino-Triazine Derivatives

**Figure 5:**
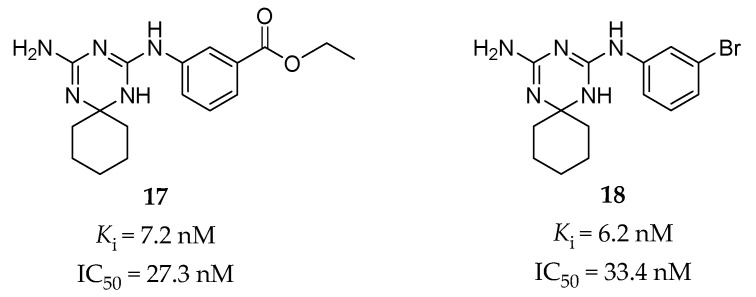
Representative triazine derivatives as selective 5-HT_2B_R antagonists [65].

Using the structure-based hierarchical virtual screening approach [133], Huang et al. have identified a series of bis-amino-triazine derivatives as potent 5-HT_2B_R antagonists [65]. Two compounds (compound **17** and **18**, Figure 5) were highlighted with comparable potency in the binding and functional assays in vitro (**17**: *K*_i_ = 7.2 nM, IC_50_ = 27.3 nM in calcium flux assay; **18**: *K*_i_ = 6.2 nM, IC_50_ = 33.4 nM in calcium flux assay). Without the classical tertiary amine group, these compounds performed good binding selectivity for the 5-HT_2B_R over ten other tested 5-HT receptors [65]. In vivo studies further indicated that compound **18** could significantly attenuate visceral hypersensitivity in an IBS rat model [65].

The predicted binding mode for compound **17** was verified by SAR analysis of a series of structural analogs [65]. The key interactions contain a typical salt bridge interaction between the protonated triazine ring and the carboxyl group of the conserved D135^3.32^ residue, hydrogen bonds between one amino (NH) group on the triazine ring and the D135^3.32^ residue, between the other amino (NH2) group and the N344^6.55^ residue, and between the ethyl benzoate moiety and the T140^3.37^ residue [65]. Notably, further assessment of the binding model for compound **18** showed that its potent binding affinity might be due to a halogen bonding interaction between the bromine atom and the carbonyl oxygen atom of the F217^5.38^ residue, which was supported by the subsequent SAR study and MD simulation [134].

#### 4.2.3. Guanidine Derivatives

Inspired by the synergistic effect of the 5-HT_2B_R selective antagonist RS-127445 and the 5-HT_7_R selective antagonist SB-269970 found in the guinea pig model evaluating the antimigraine effect, Moritomo et al. discovered a series of carbonyl guanidine derivatives as dual 5-HT_2B_R and 5-HT_7_R antagonists for the treatment of migraines.

**Figure 6:**
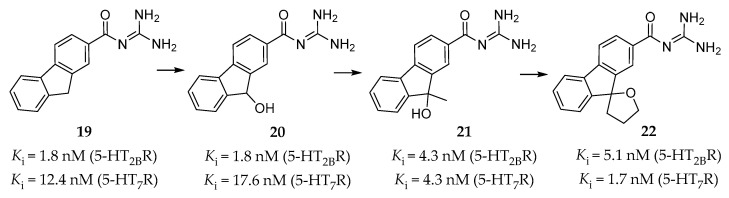
Carbonyl guanidine derivatives as dual 5-HT_2B_R and 5-HT_7_R antagonists [135,136,137].

Originally, high throughput screening (HTS) led to the discovery of compound **19** (Figure 6) [135], which showed a high affinity for the 5-HT_2B_R (*K*_i_ = 1.8 nM) and the 5-HT_7_R (*K*_i_ = 12.4 nM), but poor aqueous solubility. Further SAR studies on a series of guanidine derivatives led to the identification of compound **20** (Figure 6), with a similar binding affinity (*K*_i_ = 1.8 nM for the 5-HT_2B_R, *K*_i_ = 17.6 nM for the 5-HT_7_R) and better aqueous solubility when compared to compound **19**. The off-target assessment showed that compound **20** was selective for the 5-HT_2B_R and the 5-HT_7_R over several other monoaminergic GPCRs, and further functional assay determined its antagonistic activity towards the 5-HT_2B_R and the 5-HT_7_R [135]. Furthermore, in vivo studies showed that compound **20** had an inhibitory effect on 5-HT-induced dural PPE in guinea pigs at 3 mg/kg intraperitoneal administration. However, it was not able to reduce the amount of leaked protein from the dural blood vessel to the reference value at 30 mg/kg oral administration [135], which indicates a deficient oral bioavailability.

In order to improve oral potency and keep high affinity, the researchers further optimized guanidine **20** by taking the balance between lipophilicity and polar surface area into consideration [136]. SAR studies based on molecular modeling results led to the identification of compound **21**, which showed both high affinity (*K*_i_ = 4.3 nM for the 5-HT_2B_R, *K*_i_ = 4.3 nM for the 5-HT_7_R, Figure 6) and selectivity [136]. When orally administered at 30 mg/kg in in vivo studies, compound **21** reversed the hypothermic effect of 5-carboxamidotryptamine in mice and showed a suppressing effect to normal levels on 5-HT-induced dural PPE in guinea pigs [136].

Considering about oxidation or conjugate metabolism of 9-OH in the fluorene ring of compound **21**, a spiro cycloalkane ring was thus introduced to eliminate the concern about drug metabolism. This design led to a guanidine derivative **22** (Figure 6), which was found to be a high-affinity and selective antagonist of the 5-HT_2B_R and the 5-HT_7_R (*K*_i_ = 5.1 nM for the 5-HT_2B_R, *K*_i_ = 1.7 nM for the 5-HT_7_R) [137]. In vitro studies showed that both its optically pure isomers, (*R*)-**22** and (*S*)-**22**, exhibited similar binding affinities and antagonistic activities against the 5-HT_2B_R and the 5-HT_7_R. Moreover, they both suppressed 5-HT-induced dural PPE and the amount of leaked protein to near normal levels at 10 mg/kg, p.o. in guinea pigs [137].

#### 4.2.4. Chromone Derivatives

**Figure 7:**
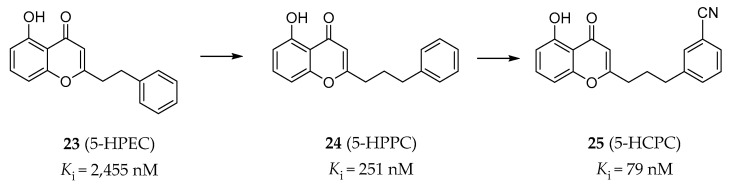
Representative chromone derivatives as non-nitrogenous 5-HT_2B_R antagonists [138,139,140].

In order to study the molecular mechanism of the neuroprotective activity of 5-hydroxy-2-(2-phenylethyl)chromone (5-HPEC) (**23**, Figure 7) [138], a natural product isolated from Imperata cylindrical, Williams and colleagues performed a screening campaign against the CNS receptors, transporters and ion channels. The results showed that 5-HPEC is a 5-HT_2B_R antagonist, as was verified in radiobinding assays (*K*_i_ = 2455 nM) and calcium flux functional assays (IC_50_ = 8913 nM). 5-HPEC showed selectivity for the 5-HT_2B_R over other 5-HT_2_Rs. A subsequent SAR study on a series of synthesized and 5-HPEC’s natural analogs was performed and showed that the most potent analog, 5-hydroxy-2-(2-phenylpropyl)chromone (5-HPPC) (**24**, Figure 7), exhibited a 10-fold improvement in the 5-HT_2B_R affinity (*K*_i_ = 251 nM) and was able to maintain the 5-HT_2B_R antagonism [139]. Recently, further optimization of 5-HPPC guided by molecular modeling approaches helped to identify 5-hydroxy-2-(3-(3-cyanophenyl)propyl)chromone (5-HCPC) (**25**, Figure 7), which exhibited an improved binding affinity (*K*_i_ = 79 nM) compared with 5-HPPC and maintained inhibitory activity (IC_50_ = 6310 nM in calcium flux assay) at the 5-HT_2B_R, as well as selectivity over the 5-HT_2A_R and the 5-HT_2C_R [140]. It is worth mentioning that these chromone derivatives are non-nitrogenous, which are different from the typical nitrogen-containing ligands of the 5-HT_2B_R. Although the binding modes of this type of ligands were predicted by molecular docking, the evidence was insufficient to effectively demonstrate that these non-nitrogenous ligands bind to the orthosteric site of the 5-HT_2B_R. Considering the relatively weak cellular activity of 5-HCPC with much stronger binding affinity, it is not possible to exclude the possibility that they represent allosteric-site binders.

#### 4.2.5. C4 Phenyl Aporphines and Tris-(phenylalkyl)amines

**Figure 8:**
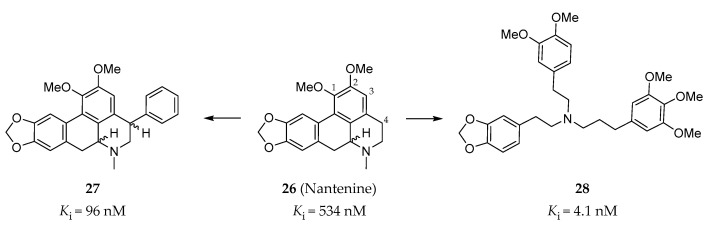
Representative C4 phenyl aporphines (**27**) and tris-(phenylalkyl)amines (**28**) as 5-HT_2B_R antagonists [142,143].

The Harding group at Hunter college focused on the synthesis and evaluation of nantenine analogs as 5-HT_2A_R antagonists. Nantenine (**26**, Figure 8) is a natural product with binding affinities to a number of CNS receptors (α_1A_R: *K*_i_ = 2 nM; 5-HT_2A_R: *K*_i_ = 850 nM, 5-HT_2B_R: *K*_i_ = 534 nM) [141]. In order to increase the 5-HT_2A_R affinity of nantenine, a series of nantenine analogs were designed. Surprisingly, nantenine analogs bearing a phenyl ring substituent at the C4 position displayed selective affinities to the 5-HT_2B_R [142]. Compound **27** (Figure 8) exhibited the best binding affinity (*K*_i_ = 96 nM) and was found to be a 5-HT_2B_R antagonist (IC_50_ = 1000 nM in calcium flux assay) with good selectivity over other tested CNS receptors [142]. In addition, in order to investigate whether the molecular rigidity of the aporphine template of nantenine affects the 5-HT_2A_R antagonism, a series of tris-(phenylalkyl)amines with increased flexibility compared with nantenine were synthesized. Similarly, these tris-(phenylalkyl)amines were found to have a high affinity and selectivity, as well as antagonist activity to the 5-HT_2B_R [143]. Among them, compound **28** (Figure 8) showed the best binding affinity to the 5-HT_2B_R (*K*_i_ = 4.1 nM, IC_50_ = 1259 nM in calcium flux assay), with a > 30-fold selectivity over the 5-HT_2A_R and the 5-HT_2C_R [143].

#### 4.2.6. Biphenyl Amide Derivatives

**Figure 9:**
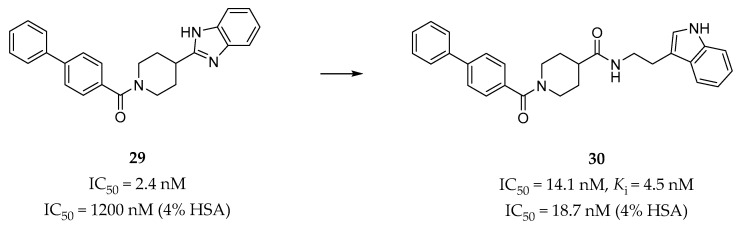
Representative biphenyl amide derivative (**30**) as 5-HT_2B_R antagonists [144].

Gabr et al. identified a series of biphenyl amide derivatives as 5-HT_2B_R antagonists by rational drug design utilizing a pharmacophore-based approach [144]. The pharmacophore map was based on a previously published doxepin induced-fit model of the 5-HT_2B_R [65]. The pharmacophore of the lead compound **29** (Figure 9) [145], a potent 5-HT_2B_R antagonist (IC_50_ = 2.4 nM in calcium flux assay) with poor potency in the presence of human serum albumin (HSA) (IC_50_ = 1200 nM in 4% HSA), was initially overlaid with the receptor-based pharmacophore in order to provide directions for optimization. Finally, compound **30** (*K*_i_ = 4.5 nM, IC_50_ = 14.1 nM in calcium flux assay, Figure 9) was identified with high potency and selectivity for the 5-HT_2B_R over six other 5-HT receptors. In vitro, pharmacokinetic profile evaluation showed that compound **30** was able to almost completely maintain its antagonistic potency in the presence of 4% HSA (IC_50_ = 18.7 nM). In terms of the predicted binding mode, compound **30** could form an additional hydrogen bond with residue N344^6.55^, and hydrophobic interactions with several residues in the ECL2 of the receptor compared with compound **29** [145]. These additional interactions were regarded as contributing to both the potency and the selectivity of compound **30**.

#### 4.2.7. Chromane Derivatives

**Figure 10:**
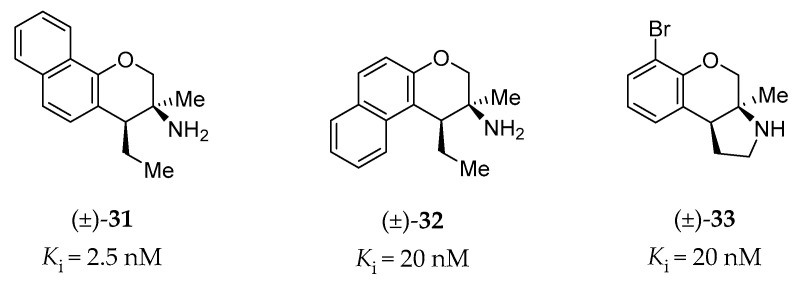
Representative chromane derivatives as 5-HT_2B_R high-affinity ligands [146].

Porter et al. have investigated the GPCR affinity for a variety of heterocyclic phenethylamine derivatives, which helped them to identify several subtype-selective ligands, including 5-HT_2B_R ligands [146]. High-affinity (<100 nM) and high selectivity (>10-fold affinity over other tested GPCRs: 5-HT_1A_R, 5-HT_7_R, σ_1_, and σ_2_ receptors) 5-HT_2B_R ligands are shown in Figure 10 (racemic compound **31**, **32** and **33**), all of which are chromane derivatives.

#### 4.2.8. Other Pharmaceutical Ligands of the 5-HT_2B_R

In 2010, a urea type of ligand **34** (*K*_i_ = 42 nM, Table 3) was coincidentally discovered as a 5-HT_2B_R antagonist by Kwon et al. [147]. Notably, this compound showed strong selectivity for the 5-HT_2B_R over other serotonin receptors, as well as dopamine, histamine, muscarinic and opiate receptors. In 2011, C-122 (**35**, Table 3) was reported by Zopf et al. as a 5-HT_2B_R antagonist (*K*_i_ = 5.2 nM) [148]. It is worth mentioning that C-122 is not selective for the 5-HT_2B_R and also exhibit binding affinities against serotonin receptors such as the 5-HT_7_R (*K*_i_ = 4.4 nM), the 5-HT_2A_R (*K*_i_ = 61 nM), and several other monoaminergic GPCRs. In vivo studies on the MCT-induced PAH rat model showed that C-122 prevented MCT-induced elevations in the pulmonary arterial circuit pressure, right ventricular hypertrophy and pulmonary arteriole muscularization when orally administered at 10 mg/kg daily for 3 weeks [148]. In 2015, Rodrigues et al. carried out a proof-of-concept study of a large-scale multidimensional de novo design approach, combining computational molecular design and quantitative activity prediction with microfluidics synthesis, and discovered new chemical entities for the 5-HT_2B_R [149]. Several computationally designed compounds with a good predicted affinity and selectivity were subjected to experimental validation. As a result, piperazine **36** (Table 3) was identified as a 5-HT_2B_R antagonist (*K*_i_ = 251 nM), with high binding and functional selectivity [149]. In 2016, considering that adenosine derivatives were reported with micromolar activity at the 5-HT_2B_R and the 5-HT_2C_R, Tosh et al. applied a structure-based drug design approach to further improve the 5-HT_2_R affinity and simultaneously reduce the affinity to adenosine receptors (ARs) [150]. A SAR study on a series of adenosine derivatives assisted them in identifying several antagonists of the 5-HT_2B_/5-HT_2C_ receptors with selectivity over ARs. Among them, compound **37** (Table 3) showed high binding affinity (*K*_i_ = 23 nM) and antagonist activity for the 5-HT_2B_R, with 12-fold binding selectivity and 170-fold functional selectivity for the 5-HT_2B_R over the 5-HT_2C_R [150]. In 2018, Rataj et al. combined a fingerprint-based machine learning approach and molecular docking that led to the identification of compound **38** (Table 3) [151]. Notably, compound **38** showed potent binding affinity (*K*_i_ = 0.3 nM) and >10,000-fold selectivity over other five tested 5-HTRs [151]. In addition, in 2018, as a proof-of-concept of halogen bonding in designing 5-HT_2B_R ligands, a series of halogen-substituted analogs of doxepin (*K*_i_ = 25.3 nM for the 5-HT_2B_R) were synthesized. As expected, the bromine-substituted compound **39** (Table 3) showed a 10-fold increased binding affinity towards the 5-HT_2B_R (*K*_i_ = 2.5 nM), a 10-fold improvement when compared to doxepin, and exhibited superior potency in a mouse model of diarrhea [134].

### 4.3. Summary of Binding Features of 5-HT_2B_R Ligands

**Figure 11:**
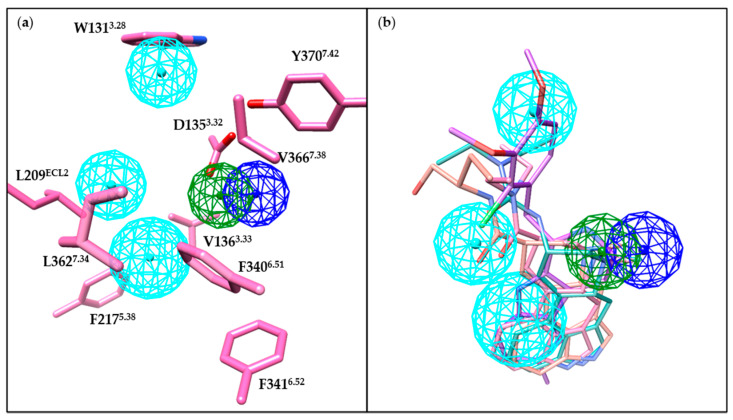
Key pharmacophore features of the 5-HT_2B_R. Positive electrostatic, hydrogen bond donor and hydrophobic pharmacophore features are colored in blue, green, and cyan, respectively. Pharmacophore features were generated based on the 5-HT_2B_R crystal structure (PDB ID: 5TVN) using the CavityPlus web server [152]. (**a**) Association of 5-HT_2B_R residues and pharmacophore features. Residues close the pharmacophore and contributing to the protein–ligand interactions are shown in hot pink sticks. (**b**) Association of co-crystal ligands and pharmacophore features. LSD (PDB ID: 5TVN), lisuride (PDB ID: 6DRX), methylergometrine (PDB ID: 6DRY), and LY266097 (PDB ID: 6DS0) are shown as hot pink, light sea green, salmon, and purple stick, respectively. These ligands are aligned based on residues in the binding pocket. Molecular images were generated using the UCSF Chimera software [115].

The crystal structures of the 5-HT_2B_R facilitate our detailed understanding of receptor-ligand binding interactions. Therefore, we generated a receptor-based pharmacophore model (Figure 11a) to reconcile the critical binding interactions of the pharmaceutical ligands mentioned above. Notably, co-crystal ligands fit well in this pharmacophore model (Figure 11b). Most of the 5-HT_2B_R ligands bear positively charged nitrogens or polar NH groups to form favorable salt bridge or hydrogen bond interaction with the carboxylic side chain of residue D135^3.32^ (Figure 11a). Moreover, aromatic rings or hydrophobic fragments of 5-HT_2B_R ligands shall form π-π stacking or hydrophobic interactions with residues in the OBP in helices III, V, VI and VII; and residues in the EBP in helices III and VII, as well as in the ECL2 (Figure 11a). Such a pharmacophore model can be expected to guide the discovery and development of new 5-HT_2B_R ligands.

## 5. Conclusions

The 5-HT_2B_R has been implicated in multiple diseases, including cardiovascular diseases, fibrosis disorders, cancer, IBS, migraine and neuropsychiatric disorders. The increasing number of crystal complex structures of the 5-HT_2B_R has provided great insights into ligand recognition, activation mechanism, and biased signaling. Nonetheless, inactive-state 5-HT_2B_R structural information and G protein and β-arrestin bound 5-HT_2B_R structures are still desirable to be obtained to further promote the development of novel 5-HT_2B_R ligands. Although many 5-HT_2B_R antagonists have been identified, with several candidates having advanced into clinical trials, no approved drug currently exists for the 5-HT_2B_R. Based on the recently solved crystal structures of GPCRs, particularly the closest homologs 5-HT_2A_R and 5-HT_2C_R [153,154,155], more research efforts should be employed to develop subtype-selective 5-HT_2B_R antagonists. Moreover, most existing 5-HT_2B_R ligands were discovered without the consideration of biased signaling, while it is desirable to study the biased signaling of the identified 5-HT_2B_R ligands, which may not only facilitate to understand the therapeutic effect-related signaling but also develop therapeutic drugs to avoid side effects.

## Figures and Tables

**Table 1:** Published 5-hydroxytryptamine 2B receptor (5-HT_2B_R) crystal structures.

PDB ID	Ligand	State	Ligand Function	Resolution (Å)
4IB4 [110]	ERG	Intermediate	β-arrestin-biased agonist	2.7
4NC3 [111]	ERG	Intermediate	β-arrestin-biased agonist	2.8
5TVN [112]	LSD	Intermediate	β-arrestin-biased agonist	2.9
5TUD [113]	ERG + antibody Fab fragment	Active	β-arrestin-biased agonist	3.0
6DRX [114]	Lisuride	Intermediate	Antagonist	3.1
6DRY [114]	Methylergometrine	Intermediate	Agonist	2.9
6DRZ [114]	Methysergide	Intermediate	Antagonist	3.1
6DS0 [114]	LY266097	Intermediate	Antagonist (G_q_ partial agonist, β-arrestin2 antagonist)	3.2

**Table 2:** 5-HT_2B_R antagonists once came into clinical trials or are in clinical trials.

Compound Name	Structure	Activity	Highest Clinical State	Indication
**7** [125] (MT-500, RS-127445)	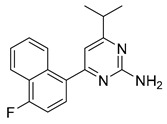	*K*_i_ = 0.3 nM	Phase 1	Migraine
**8** [126] (PRX-08066)	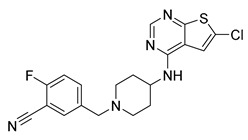	*K*_i_ ~3.4 nM	Phase 2	PH, COPD
**9** [79] (BF-1)	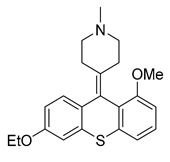	*K*_i_ = 2.7 nM	Phase 1	Migraine
**10** [127] (AMAP102)	Undisclosed	Undisclosed	Phase 2	Osteoarthritis
**11** [128] (AM-1030)	Aminoguanidine derivative	*K*_i_ = 330 nM	Phase 2	AD
**12** [64] (RQ-00310941)	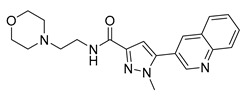	*K*_i_ = 2.0 nM	Phase 1	UC

**Table 3:** Other representative pharmaceutical ligands of the 5-HT_2B_R.

Compound Name	Structure	Activity Data
**34** [147]	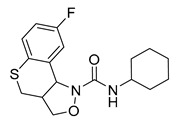	*K*_i_ = 42 nM
**35** [148] (C-122)	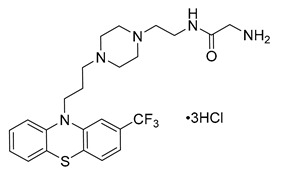	*K*_i_ = 5.2 nM
**36** [149]	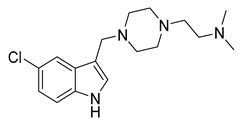	*K*_i_ = 251 nM
**37** [150]	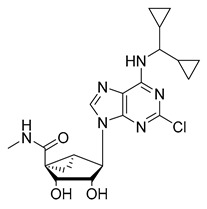	*K*_i_ = 23 nM
**38** [151]	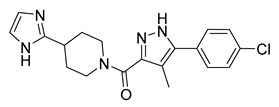	*K*_i_ = 0.3 nM
**39** [134]	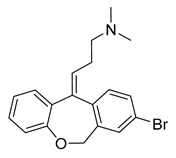	*K*_i_ = 2.5 nM

## Abbreviations

5-HT_2B_R, 5-hydroxytryptamine 2B receptor; 5-HT, 5-hydroxytryptamine; GPCRs, G protein-coupled receptors; 5-HTRs, 5-HT receptors; 5-HT_2_R, 5-HT_2_ receptor; GTP, guanosine triphosphate; GDP, guanosine diphosphate; PLCβ, phospholipase Cβ; DAG, diacylglycerol; IP_3_, inositol triphosphate; VHD, valvular heart disease; PAH, pulmonary arterial hypertension; VICs, valvular interstitial cells; ECM, extracellular matrix; MDMA, 3,4-methylenedioxymethamphetamine; BM, bone marrow; MVP, mitral valve prolapse; CAVD, calcific aortic valve disease; DOCA, deoxycorticosterone acetate; MCT, monocrotaline; PH, pulmonary hypertension; IPF, idiopathic pulmonary fibrosis; BLM, bleomycin; TGF-β1, transforming growth factor β1; SSc, systemic sclerosis; tsk-1, tight-skin-1; HADF, human adult dermal fibroblasts; ICC, interstitial cells of Cajal; PLC, phospholipase C; PKCγ, protein kinase Cγ; IBS, irritable bowel syndrome; NO, nitric oxide; mCPP, meta-chlorophenylpiperazine; PPE, plasma protein extravasation; CNS, central nervous system; SSRIs, serotonin-specific reuptake inhibitors; DA, dopamine; NAc, nucleus accumbens; mPFC, medial prefrontal cortex; GI, gastrointestinal; ERG, ergotamine; LSD, lysergic acid diethylamine; RMSD, root-mean-square deviation; OBP, orthosteric binding pocket; EBP, extended binding pocket; SAR, structure–activity relationship; MD, molecular dynamics; UC, ulcerative colitis; AD, atopic dermatitis; HTS, high throughput screening; p.o., per os; 5-HPEC, 5-hydroxy-2-(2-phenylethyl)chromone; 5-HPPC, 5-hydroxy-2-(2-phenylpropyl)chromone; 5-HCPC, 5-hydroxy-2-(3-(3-cyanophenyl)propyl)chromone; ARs, adenosine receptors.
